# Posterior Ring Apophyseal Fracture (PRAF) in a 13-Year-Old Adolescent Girl Treated With Unilateral Biportal Endoscopy: A Case Report

**DOI:** 10.7759/cureus.69351

**Published:** 2024-09-13

**Authors:** Ashwinkumar Khandge, Amit Kale, SomiReddy Medapati, Pankaj Sharma, Ketan Kulkarni, Rishyendra Varma

**Affiliations:** 1 Orthopaedics, Dr. D. Y. Patil Medical College, Hospital and Research Centre, Dr. D. Y. Patil Vidyapeeth (Deemed to be University), Pune, IND

**Keywords:** adolescent, low back pain, lumbar disc herniation, minimally invasive surgery, posterior ring apophyseal fracture, unilateral biportal endoscopy

## Abstract

Posterior ring apophyseal fracture (PRAF) is a rare but significant cause of lower back pain and radiculopathy in adolescents, commonly occurring due to trauma or intense physical activity. This is a case report of a 13-year-old girl with PRAF associated with lumbar disc herniation (LDH) following a sports injury, emphasizing diagnostic challenges and surgical management. The patient underwent unilateral laminectomy for bilateral decompression through unilateral biportal endoscopy (UBE-ULBD) at the L4-L5 level. Postoperatively, the patient had significant clinical improvement in the visual analog scale (VAS) score for leg and back pain at one-month follow-up. Although rare, PRAF associated with LDH is an important differential diagnosis for post-traumatic lower back pain in adolescents. Accurate preoperative imaging and minimally invasive surgical techniques such as UBE-ULBD provide effective decompression and improved clinical outcomes, allowing for a quicker return to daily activities.

## Introduction

Spinal injuries in the pediatric age group are about 1%-10% of all traumatic spinal injuries [1]. The terms "apophyseal ring fracture," "posterior ring apophysis separation," "lumbar posterior marginal node," "lumbar posterior marginal node," "posterior ring apophysis fracture (PRAF)," "fracture of the vertebral limbus," and "posterior Schmorl's node" have all been applied to a tiny bony fragment that is occasionally found next to the vertebral body posterior corner at the cephalad or caudal edge in the lower lumbar spine [2,3]. It is seen where the ring apophysis does not completely fuse with the vertebral body beside it before the age of 18-25 years [3]. The incidence of PRAF is about 5.7% among all patients with lumbar disc herniation (LDH) [2]. It is a rare illness usually discovered in adolescents and young adults, particularly in young, avid athletes, and for the same reason, it is more common in young males.

PRAF is becoming more widely acknowledged nowadays as a cause of neurological symptoms and low back pain [4]. PRAF can increase the risk of deformity of the spine in growing children [5]. To diagnose, a high index of suspicion, a thorough history review, a physical examination, and computed tomography (CT) are required [6].

Literature has little information about the clinical and radiological features, appropriate surgical approaches, and outcomes of PRAF associated with LDH in the adult population, despite certain studies suggesting that this association may occur in patients much older than we previously thought [2]. There are many contrasting opinions about the most appropriate decompressive techniques, whether or not to perform discectomy or apophyseal fragments, and whether or not further spinal fusion is required. However, treatment options for PRAF with LDH have not yet been thoroughly studied [3]. This case report aims to draw attention to PRAF, associated with LDH, a rare condition occurring in adolescents and young adults, to provide a consensus on one of the treatment options [7].

## Case presentation

This is a case report of a 13-year-old adolescent girl patient with low back pain following an alleged history of a fall during a sports activity three months back with a neurogenic claudication distance of 200 meters. The patient was having a tingling sensation over the posterior aspect of bilateral lower limbs. On examination, the straight leg raise (SLR) test was positive at 50 degrees on the left and 60 degrees on the right lower limb. The patient did not have any neuro deficit, and there was no bowel and bladder involvement/incontinence. The patient did not have any other associated comorbidities. The visual analog scale (VAS) score is 2/10 for back pain and 7/10 for leg pain. No significant improvement was seen with conservative treatment.

**Figure 1:**
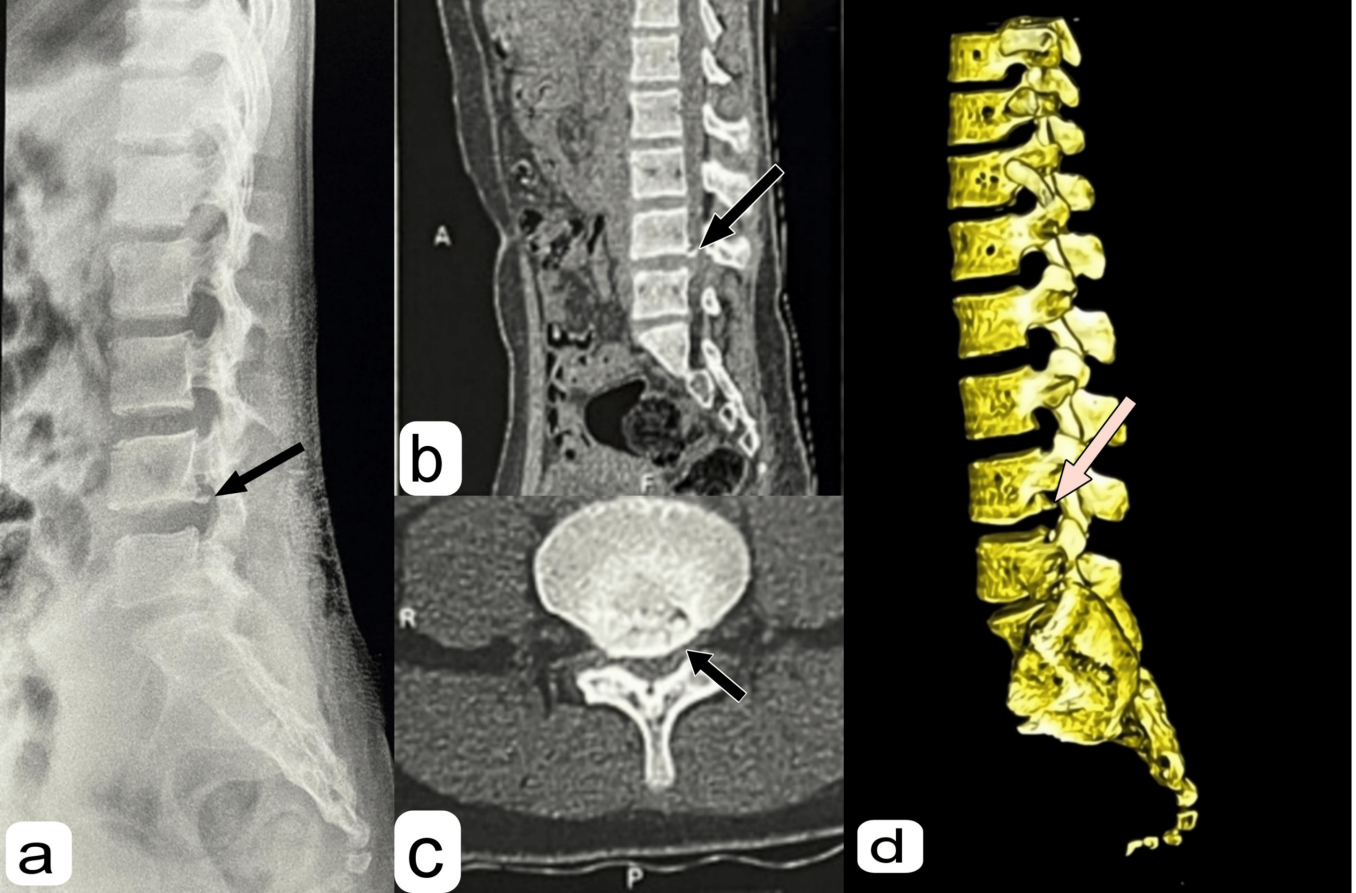
(a) X-ray of the LS spine lateral view, (b) sagittal section of CT of the LS spine, (c) axial view of L4 CT, and (d) 3D CT of the LS spine (a) X-ray of the LS spine lateral view shows a PRAF fragment at the posterior caudal end of the L4 vertebra (black arrow). (b) The sagittal section of the CT of the LS spine shows a PRAF fragment (black arrow). (c) The axial section of the CT at L4 shows the PRAF fragment (black arrow). (d) 3D CT of the LS spine showing the PRAF fragment (pink arrow). LS: lumbosacral, PRAF: posterior ring apophyseal fracture, CT: computed tomography

**Figure 2:**
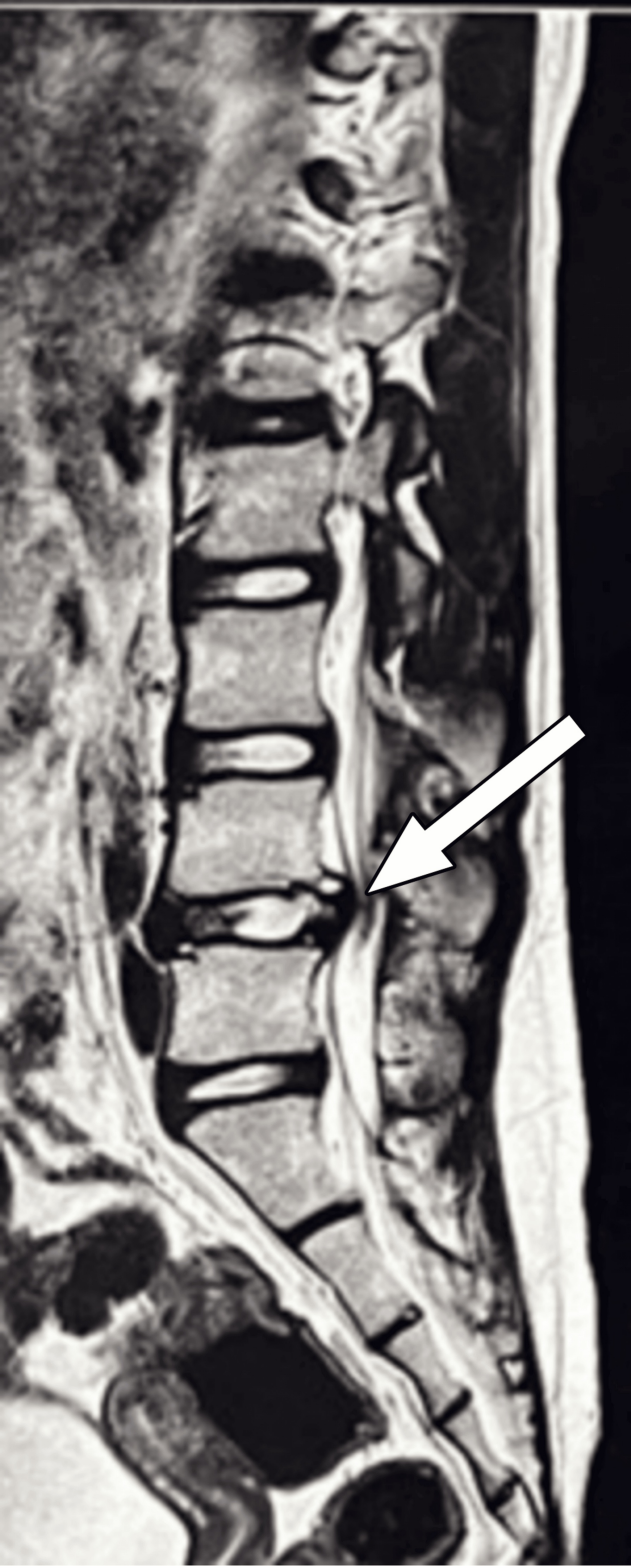
Sagittal section of the MRI of the LS spine The sagittal section of the MRI of the lumbosacral spine showing partial disc desiccation with diffuse disc bulge at the L4-L5 level (white arrow). MRI: magnetic resonance imaging, LS: lumbosacral

**Figure 3:**
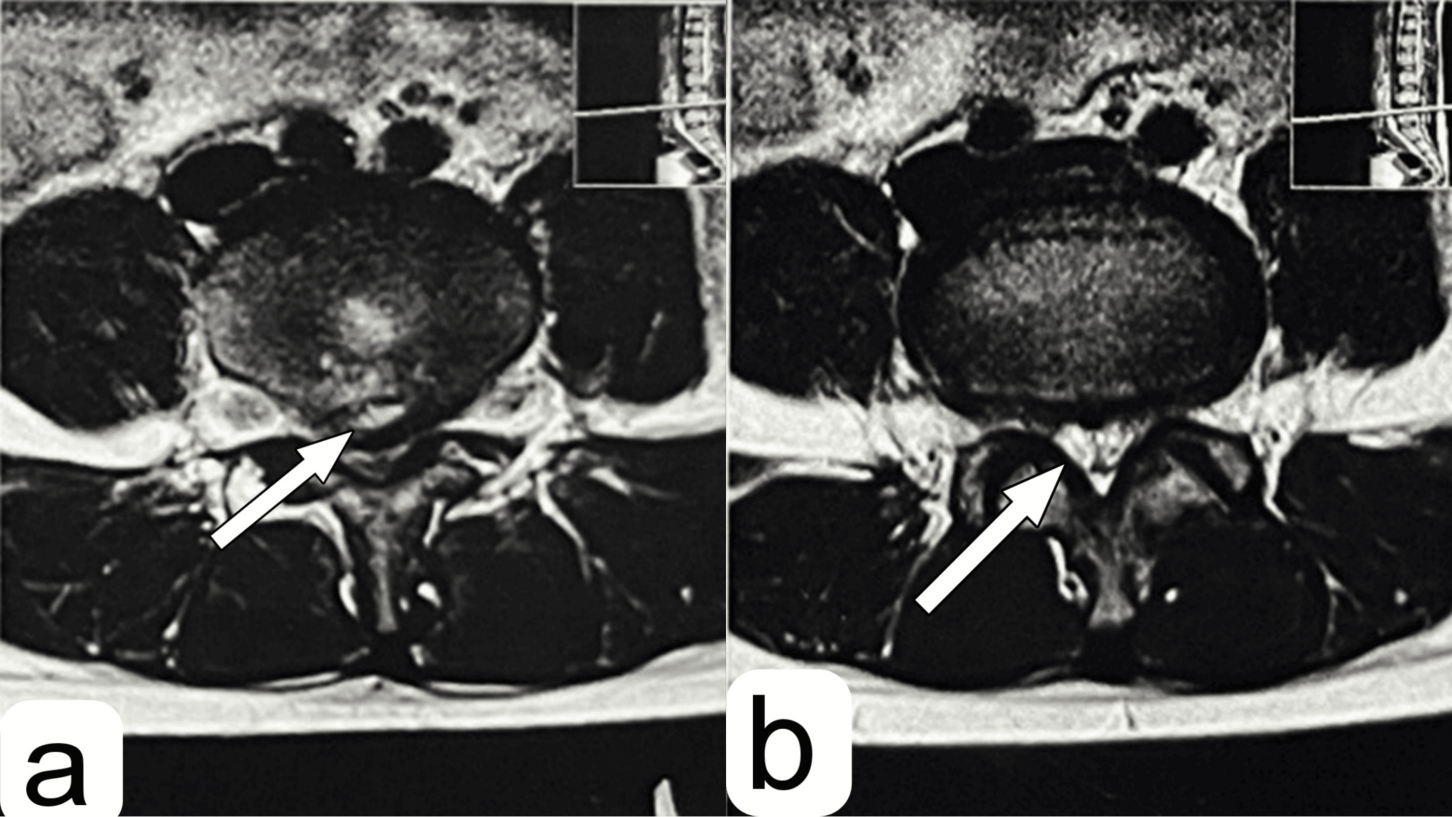
T2-weighted MRI axial sections of the L4-L5 level T2-weighted MRI axial cut showing (a) posterior ring apophyseal fracture fragment (white arrow) and (b) severe compromise of bilateral lateral recesses, ligamentum flavum hypertrophy with compression of bilateral L5 traversing, and abutment of bilateral L4 exiting nerve roots due to mild compromise of bilateral neural foramina (white arrow). MRI: magnetic resonance imaging

A preoperative radiological investigation was performed. X-ray suggested a PRAF at the caudal end of the L4 vertebra (Figure 1a). To confirm the diagnosis, CT was performed, and the fragment was identified (Figure 1b-1d). Magnetic resonance imaging (MRI) of the lumbosacral spine was performed to investigate any compression over the cord or the traversing and exiting nerves. It showed partial disc desiccation with diffuse disc bulge at L4-L5 level (Figure 2) with mild ligamentum flavum hypertrophy, causing severe compromise of bilateral lateral recesses, with compression of bilateral L5 traversing and abutment of bilateral L4 exiting nerve roots due to mild compromise of bilateral neural foramina (Figure 3).

Surgical technique

We performed unilateral laminectomy for bilateral decompression by unilateral biportal endoscopy (UBE-ULBD) at the L4-L5 level. The patient was taken into a prone position after successful general anesthesia onto the operative table with a Wilson frame on it. After the scrubbing, painting, and draping, using C-Arm fluoroscopy, L4-L5 was identified and marked using an 18G needle. The surgeon was standing on the patient's left side. A vertical mid-spinous process line and one more vertical line, 1-1.5 cm lateral to the midline, were drawn, and triangulation was done to reach the spinus process-lamina junction of L4 on the left side. The caudal portal was the instrumentation/working portal, and the cranial portal was the viewing/camera portal. A radio frequency (RF) wand was used to ablate soft tissue to expose the spino-laminar surface. Using a high-speed diamond burr, we burred out the inferior part of the L4 lamina up until the ligamentum flavum attachment, which is the usual uppermost aspect of the lumbar canal stenosis at that level, and some part of the base of the spinous process, to visualize the contralateral ligamentum flavum.

**Figure 4:**
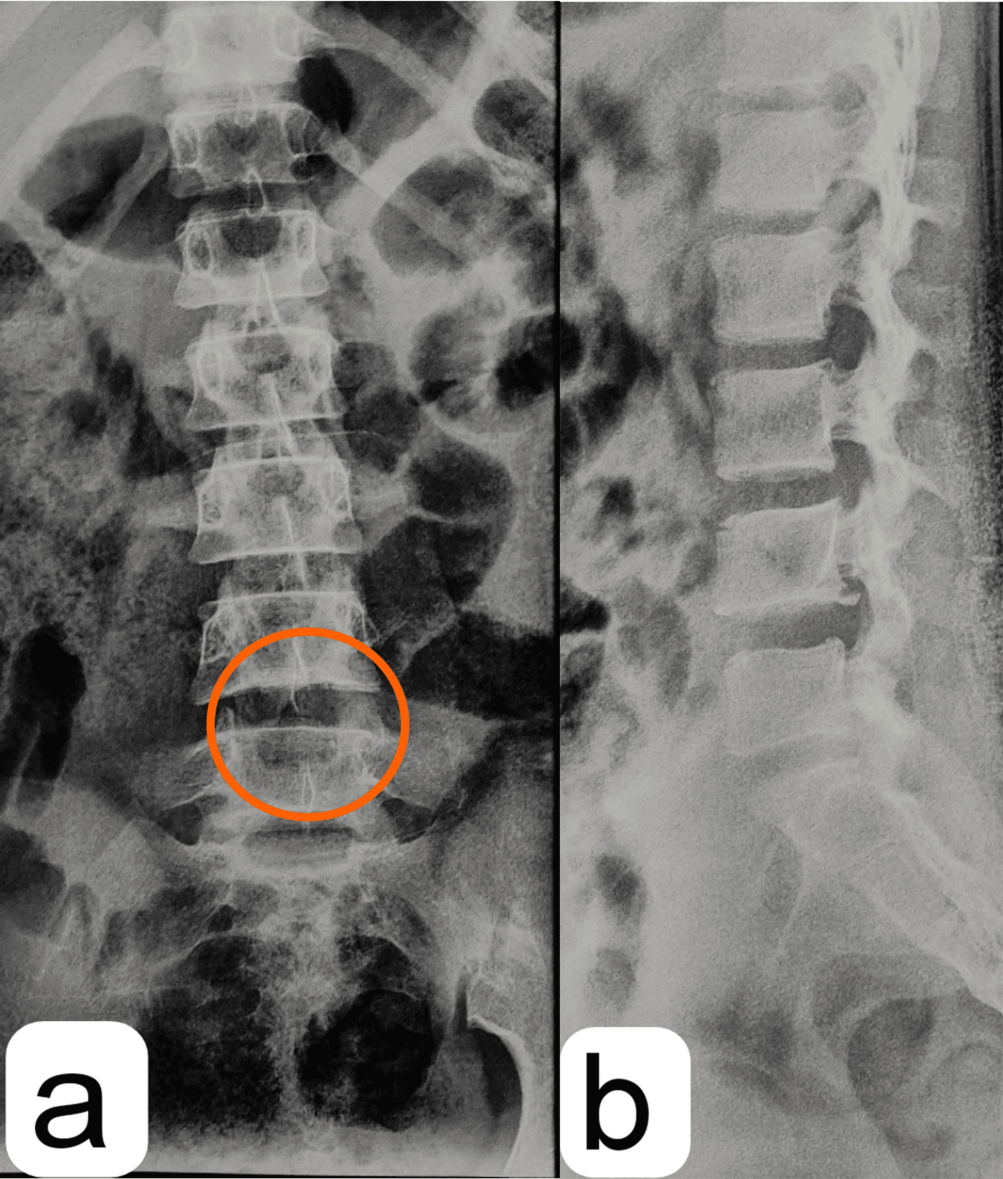
Postoperative X-ray (a) AP and (b) lateral view of the LS spine Postoperative X-ray showing the (a) AP view of the LS spine with the site of laminectomy and bilateral decompression at the L4-L5 level on the left side (marked area) and (b) postoperative X-ray of the LS spine in lateral view. AP: anteroposterior, LS: lumbosacral

Diamond burr and Kerrison rongeurs of size 2 and 3 mm were used to decompress contralaterally by sublaminar decompression. Curved curet is used to free the ligamentum flavum from under the surface of the caudal lamina. The ballpoint nerve hook adhesions between the flavum and the dura below are removed to prevent complications such as durotomy before flavectomy. Flavectomy is done using Kerrison rongeurs, and the medial facet was burred out to expose the L5 nerve root, which is traversing. The superior part of the inferior lamina is removed using burr and Kerrison rongeurs to decompress the inferior part (Figure 4). A curet is used to detach the ligamentum flavum attachment on the contralateral side. A nerve hook is used to clear the attachments between the dura and ligamentum flavum carefully. The flavum was removed to expose the contralateral recess and traversing nerve root (Video 1). The whole procedure was uneventful, and the decompression looked sufficient as the nerve roots were free. The L4-L5 disc was palpated using a probe, which was hard, so the discectomy was not performed. Closure and then aseptic dressing was done.

**Video 1:** UBE-ULBD steps Video showing the UBE-ULBD steps performed in this patient. UBE-ULBD: unilateral laminectomy for bilateral decompression by unilateral biportal endoscopy

Postoperatively, the patient was comfortable and able to walk without claudication pain. The patient's VAS score of leg pain has improved postoperatively to 3/10 immediately postoperatively and 1/10 at one-month follow-up. The VAS score of back pain was 2/10 immediately postoperatively and 0/10 at one-month follow-up, and the straight leg raise has improved to 80 degrees bilaterally.

## Discussion

A rare but significant cause of lower back pain in adolescents is a PRAF, which typically occurs after a trauma or intense physical activity such as athletics [7]. At around five years old, the ring apophyses appear and usually fuse with the remaining part of the vertebral body between 18 and 20 years old, sometimes till 25 years old. Recent studies indicate that the prevalence of PRAF in pediatric disc herniations varies between 19% and 42% [8]. The incidence of PRAF varies significantly across different age groups. Among all patients with LDH, PRAF is reported to occur in 5.35%-8.2%. Its frequency ranges widely from 5.8% to 28% in children and adolescents. In comparison, the incidence is lower in adults [3]. The study by Takata et al. found that patients with PRAF are usually in their second decade of life or in their 20s but can also occur in patients in their 30s, 40s, and rarely in their 50s or older [9]. Other studies by Shirado et al. [10] and Scarfò et al. [11] reported similar findings with patients ranging in age from younger than 20 to 69 years, with the mean age typically falling between 13.5 and 36.2 years. When conservative treatment is tried, due to the bone compression, often, the condition does not improve. Surgery is recommended when conservative therapy is deemed inadequate [3].

The annulus fibrosus is attached to the superior and inferior vertebral plateaus via Sharpey's fibers and to some posterior longitudinal ligament fibers. Consequently, fractures are commonly observed along the posterior aspect and in the midline. However, they can also be seen on the superior end plate in the posterolateral area in rare cases [7,12]. An analysis of existing literature indicates that fractures most commonly affect the L4, L5, and S1 vertebral end plates, with the L5-S1 and L4-L5 levels being the most frequent locations for PRAF [3,7].

PRAF bony fragment's small size and lack of familiarity with the condition are primarily confounded with a posterior longitudinal ligament calcification or herniated disc calcification. With osteophytes of the posterior degenerative ridge, the lesion is commonly overlooked on X-ray fluoroscopy and CT [2]. CT is the diagnostic modality of choice for PRAF. It helps to differentiate the calcified or non-calcified fractures from the herniation of the lumbar intervertebral disc. MRI is used to find out the prolapsed intervertebral disc and other structures causing compression over the spinal cord and nerves [7]. Surgeons may encounter challenges with this condition due to their limited familiarity, often mistaking it for other spinal issues such as ossification of the posterior longitudinal ligament, calcification of intervertebral discs, or osteophytes along the posterior degenerative ridge [2].

Several classifications exist for categorizing these fractures, such as the Epstein classification, a modified Takata classification, widely adopted based on fracture morphology. Initially, PRAF was classified by Takata et al. into three types using CT scans [9]. Additionally, Epstein et al. proposed a rare type, type 4, which involves a larger fracture found extending from the superior and inferior endplates [13]. Our case is type II according to this classification. The aim is to relieve the symptoms of the patient in PRAF with LDH with minimal surgical intervention so that the patient can return to her activities of daily living sooner [3,4].

Given the rarity of this condition, the literature features limited case reports and lacks clear treatment guidelines [3]. While many studies recommend the removal of PRAF fragments, Shirado et al. [10] and Laredo et al. [14] have reported that fragment removal does not significantly impact clinical outcomes. The decision to remove fragments should be based on their mobility, size, location, and whether they are symptomatic for the patient [3]. Some authors suggest that discectomy is unnecessary in children without abnormal MRI findings or degenerative changes in the intervertebral discs, as observed in adults [3]. However, these studies have limitations in quality, and more extended follow-up periods are needed to assess outcomes thoroughly.

Spinal fusion is not routinely performed and is primarily indicated in cases of segmental instability when multiple or bilateral laminectomy is required or when extended facetectomy has been performed for decompression [3]. In adolescent patients, the primary goal of UBE-ULBD is neural decompression, with an emphasis on minimizing surgical trauma, reducing associated complications, and promoting rapid recovery to allow a swift return to educational or physical activities. In our case, a unilateral laminectomy was performed for bilateral decompression. The PRAF fragments were stable, and the intervertebral disc was firm without protrusion, so a discectomy was not necessary. Additionally, there was no evidence of segmental instability, so spinal fusion was not performed.

## Conclusions

This case report underscores the significance of recognizing PRAF in the pediatric population, particularly in association with LDH. Despite its rarity, PRAF can lead to substantial neurological symptoms and low back pain, necessitating a careful diagnosis involving a high index of suspicion, detailed history, physical examination, and imaging modalities such as CT and MRI.

Our case illustrates the successful management of PRAF associated with LDH through UBE-ULBD. This minimally invasive technique proved effective in alleviating symptoms and promoting recovery, with favorable outcomes and no need for additional spinal fusion. The choice to forgo discectomy in the presence of a firm, non-protruded disc and to avoid spinal fusion due to the absence of segmental instability reflects a tailored approach based on the patient's specific condition. Further studies with larger sample sizes and extended follow-up are needed to refine treatment strategies and improve outcomes for patients with PRAF and LDH.
